# Efferent pathways from the suprachiasmatic nucleus to the horizontal limbs of diagonal band promote NREM sleep during the dark phase in mice

**DOI:** 10.1186/s12868-024-00881-0

**Published:** 2024-07-22

**Authors:** Lei Chen, Changfeng Chen, Qiaoling Jin, Yue Liang, Jian Wu, Pingping Zhang, Juan Cheng, Liecheng Wang

**Affiliations:** 1 Departments of Pharmacy, The First Affiliated Hospital of Anhui University of Chinese Medicine, Hefei, 230031 China; 2 Department of Physiology and Functional Experiment center, School of Basic Medical Sciences, Anhui Medical University, Hefei, 230032 China; 3 Department of Neurology, Mayo Clinic, Rochester, MN 55905 USA

**Keywords:** Suprachiasmatic nucleus, Basal forebrain, Sleep–wake, Non–rapid eye movement sleep

## Abstract

The regulation of circadian rhythms and the sleep–wake states involves in multiple neural circuits. The suprachiasmatic nucleus (SCN) is a circadian pacemaker that controls the rhythmic oscillation of mammalian behaviors. The basal forebrain (BF) is a critical brain region of sleep–wake regulation, which is the downstream of the SCN. Retrograde tracing of cholera toxin subunit B showed a direct projection from the SCN to the horizontal limbs of diagonal band (HDB), a subregion of the BF. However, the underlying function of the SCN–HDB pathway remains poorly understood. Herein, activation of this pathway significantly increased non–rapid eye movement (NREM) sleep during the dark phase by using optogenetic recordings. Moreover, activation of this pathway significantly induced NREM sleep during the dark phase for first 4 h by using chemogenetic methods. Taken together, these findings reveal that the SCN–HDB pathway participates in NREM sleep regulation and provides direct evidence of a novel SCN-related pathway involved in sleep–wake states regulation.

**Supplementary Information:**

The online version contains supplementary material available at 10.1186/s12868-024-00881-0.

## Introduction

The sleep–wake states is mainly regulated by homeostasis and circadian rhythms [1]. In mammals, these two aspects are functionally interconnected with each other [2]. The circadian system consists of a series of central and peripheral oscillators, which allows the body to adapt to different functional demands and environments, such as sleep–wake states [3, 4]. The suprachiasmatic nucleus (SCN), which is an important circadian pacemaker in mammals, is located in the hypothalamus posterior the optic chiasm [5, 6].

Previous studies have shown that light information from intrinsically photosensitive retinal ganglion cells (ipRGCs) projects to the the SCN [7, 8]. The SCN then relays the information downstream to other nuclei for various functions, one of which is sleep–wake regulation [9, 10]. Current research shows that the SCN can regulate arousal by manipulating downstream arousal-related nuclei. For example, transsynaptic retrograde tracing and neurophysiological experimental methods have confirmed that the circuit of SCN–dorsomedial hypothalamus (DMH)–locus coeruleus (LC) regulates the light-dark difference of impulse activity [11]. Similarly, microinjection of retrograde tracers and single-unit extracellular recording showed that an indirect pathway from the SCN to the ventral tegmental area (VTA) takes part in the regulation of arousal [12]. On the aspects of sleep regulation, studies have shown that SCN neurons populations are necessary and sufficient for darktime sleep, but have no effect on lighttime sleep [13]. However, the relevant evidence associated with downstream nuclei in sleep regulation is still insufficient [14].

Studies have shown that the primary projection targets of the SCN are mainly in the hypothalamus, the thalamic nuclei and the basal forebrain (BF) [15, 16]. The BF is a large heterogeneous structure regulating sleep–wake states that contains many subregion, including the magnocellular preoptic nucleus (MCPO), substantia innominata (SI), nucleus of the horizontal limb of the diagonal band (HDB), nucleus of the vertical limb of the diagonal band (vDB), and medial septal nucleus (MS) [17, 18]. The BF is involved in modulating sleep–wake transitions, increasing arousal levels and regulating NREM sleep [19]. However, direct evidence that the SCN–BF pathway regulates sleep–wake cycles remains unknown. Therefore, we hypothesized that the pathway of SCN–BF may play a critical role in sleep–wake states regulation.

## Materials and methods

### Animals

C57BL/6J mice (7–9 w, purchased from Henan Skobes Biotechnology Co., LTD)/ Vgat-cre mice (7–9 w, purchased from Jackson Lab, 028862) were housed under a 12 h:12 h light/dark cycle (lights on at 8:00 am (ZT0), lights off at 20:00 pm (ZT 12)). Food and water were available ad libitum. All experiments were approved by the Experimental Animal Ethics Committee of Anhui Medicine University, and adhered to the guidelines of the Institutional Animal Care Unit Committee of Anhui Medicine University (project number: LLSC20190763). All methods were carried out in accordance with relevant guidelines and regulations. In our study, the injection site of each experimental animal needed to be verified after the experiment. The experimental animals used were all infused with 4% paraformaldehyde for cardiac perfusion and histological examination. Specific procedures are described in the Histology section below. The carcasses of experimental animals are sent to the animal center for unified incineration. All the experimental operation was carried out under the condition of 25–30℃. The mice were placed on an electric blanket to maintain body temperature during the procedure. Breathing is monitored by observing the fluctuating movements of the thorax.

### Anterograde and retrograde tracing

2 µg/µl cholera toxin subunit B-488 (Cat #: C34775, Thermo Fisher Scientific) or AAV2/9-hEF1a-DIO-eGFP was injected to HDB (Anteroposterior (AP): 0.22 mm; Mediodorsal (ML): 0.9 mm; Dorsoventral (DV): 5.62 mm; 10 nl, 50 nl/min) or SCN (AP: 0.46 mm; ML: 1.20 mm; DV: 5.70 mm; 10°; 50 nl, 50 nl/min). The Anterograde or retrograde signal from the tracer became detectable after 3 w or 3–7 d. Data from animals where the tracer was not restricted to the HDB were excluded. All adeno-associated virus (AAV) used in the experiment had a titer of 1.61E + 13 v.g /mL.

### Optogenetic experiments

AAV2/9-hEF1a-hChR2 (H134R)-eGFP (Cat #: S0283-9, Taitool, Shanghai) or AAV-hEF1a-eYFP (Cat #: PT0098, BrainVTA, Wuhan) was injected into the SCN. An optical fiber (0.2 mm diameter, Anilab) was inserted with the tip 0.1 mm above the HDB. Electrode for EEG/EMG recording implanted into the skull surface. After 2–3 w, the mice were familiarized with the environment for 1 w. The experiments were performed during ZT0-2 or ZT12-14 by using1 Hz, 5 Hz, 10 Hz and 20 Hz blue laser (473 nm) pulse trains (10 ms per pulse). Each trial pulse train lasted for 300 s (6–8 mW at the fiber tip, SLOC, Shanghai). Data from animals where the tracer was not restricted to the SCN and where the tip of the optical fiber was not restricted to the HDB were excluded.

### Chemogenetic experiments

AAV2/2-hSyn-Retro-tdTomato-iCre (Cat #: S0509-2R, Taitool, Shanghai) was injected into the HDB. AAV2/9-hSyn-DIO-HM3D(Gq)-eGFP (Cat #: S0260-9, Taitool, Shanghai) was injected into the SCN. Electrode of EEG/EMG was secured to the skull using dental cement. After 2–3 w, the AAV2/2-hSyn-Retro-tdTomato-iCre in the HDB caused expression of Cre recombinase in HDB-projecting neurons. Therefore, Cre recombinase allowed the expression of hM3Dq in SCN neurons projecting to the HDB following injection of DIO-hM3D(Gq)-eGFP in the SCN. The SCN–HDB pathway was activated when clozapine N-oxide (CNO) (0.1 ml/kg, Biochemicals for Life Science Research, IP) was given. Data from animals where virus expression was not restricted to the HDB and SCN were excluded. A total of 72 h were recorded. Record first 24 h as the baseline, followed by an injection of saline (0.9%, IP) at ZT 0 (high sleep drive) or ZT12 (high activity) and recording for another 24 h; then injected with CNO at ZT0 or ZT12 and recording for last 24 h.

### EEG/EMG recordings and analysis

Mice were anesthetized with pentobarbital sodium (2%). EEG/EMG recording were collected from two screws (No. 1: 1 mm anterior to bregma, ML: 1.5 mm; No. 2: 1 mm anterior to lambda, ML: 1.5 mm). Two stainless EMG wires (Cooner Wire, USA) were inserted into the left and right neck muscles respectively. EEG/EMG signals were amplified and filtered (EEG, 0.5 to 25 Hz; EMG, 20 to 200 Hz) digitalized with a sampling rate of 125 Hz (BIOPAC). The EEG and EMG data were recording by Acqknowledge 4.3. Recordings data were automatically scored offline by SleepSign as wakefulness, NREM sleep, or REM sleep in 4 s epochs using SleepSign according to standard criteria. Manually check the defined sleep-wake state and manually correct it if necessary. Different sleep-wake states were defined according to EEG and EMG waveform characteristics: Wake was defined as asynchronous, low-amplitude EEG rhythm and increased myoelectric activity accompanied by periodic bursts, which were dominated by theta waves (4–8 Hz); NREM sleep refers to synchronous, high-amplitude, low-frequency (0.5–4 Hz) delta wave dominated brain electrical activity, and EMG activity was lower than that of the awake state accompanied by bursts of phases. REM sleep was defined as having a distinct θ (4–10 Hz) rhythm, almost no myoelectric activity.

### Histology

Mice were deeply anesthetized with isoflurane and transcardially perfused with saline (0.9%) followed by 4% paraformaldehyde (PFA). The brains were incubated in 4% PFA overnight. For cryoprotection, brains were stored in 20% and then 30% sucrose (w/v) in PBS solution for at least 1 night. Brains were sliced into 40 μm sections using a frozen slicing machine (CM3050S, Leica). Fluorescence images were taken using a confocal microscope (LSM 880 + airyscan, Zeiss).

### Statistical analysis

The data are presented as the mean ± standard error of the mean (SEM). Statistical analysis was performed using Prism 7.0 (GraphPad Software). The software that was used to analyze EEG and EMG was SleepSign. One-way ANOVA were used to analyze NREM sleep, rapid eye movement (REM) sleep, wakefulness time and percentage in optogenetic experiments. One-way ANOVA and paired T test were used to analyze latency number of episodes and duration of episodes in chemogenetic experiments. Data from animals with incorrect injection sites was excluded.

## Results

### The HDB is a downstream target nucleus of the SCN

**Fig. 1:**
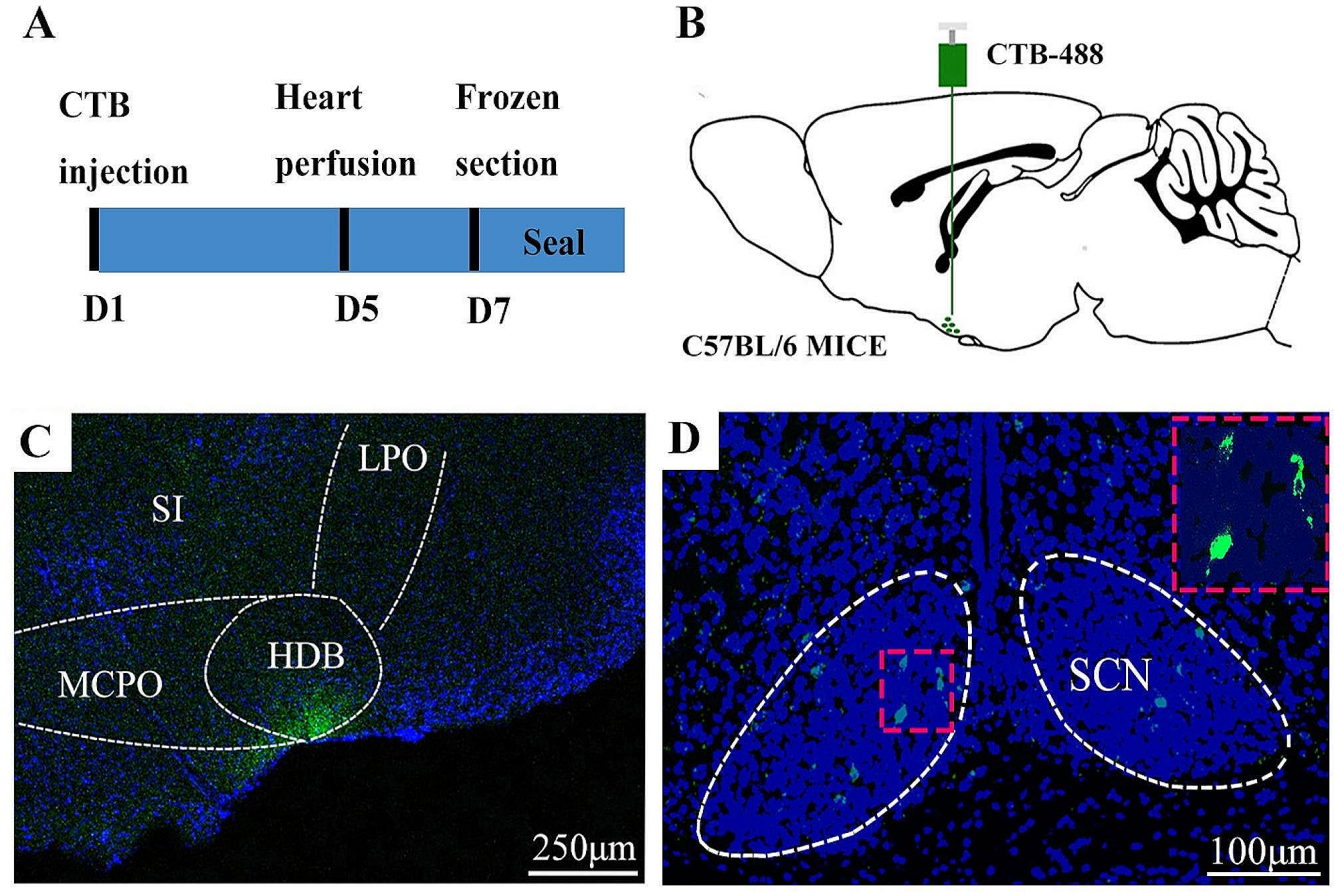
The HDB is downstream of the SCN. **A** A simple diagram of the experimental procedure. **B** Schematic of the injection of cholera toxin subunit B-488 into the HDB of C57BL/6J mice. **C** Localization of cholera toxin subunit B-488 in the HDB. The HDB, MCPO and SI are subregions of the BF. The scale bar represents 250 μm. **D** The cell bodies of neurons were labeled in the SCN. The upper right is an enlargement of the area within the red dotted square in the center. The scale bar represents 100 μm

The BF is a large, heterogeneous structure with different subregions. Previous studies have shown that BF was the downstream of the SCN, but the specific subregion was not clear enough [15, 16]. In our study, a anterograde Cre-dependent virus AAV-DIO-chR2-eGFP was injected into the SCN nucleus in Vgat-cre mice. We observed nerve fiber terminals in the HDB subregion of BF (Fig.S1). Then the retrograde tracer cholera toxin subunit B was micro-injection into the HDB subregion (Fig. 1A-C), the sparse cell bodies was appeared in SCN (Fig. 1D). The results indicated that the HDB was one of the downstream target nuclei of the SCN.

### Optogenetic activation of the SCN–HDB pathway promotes NREM sleep during the dark phase

**Fig. 2:**
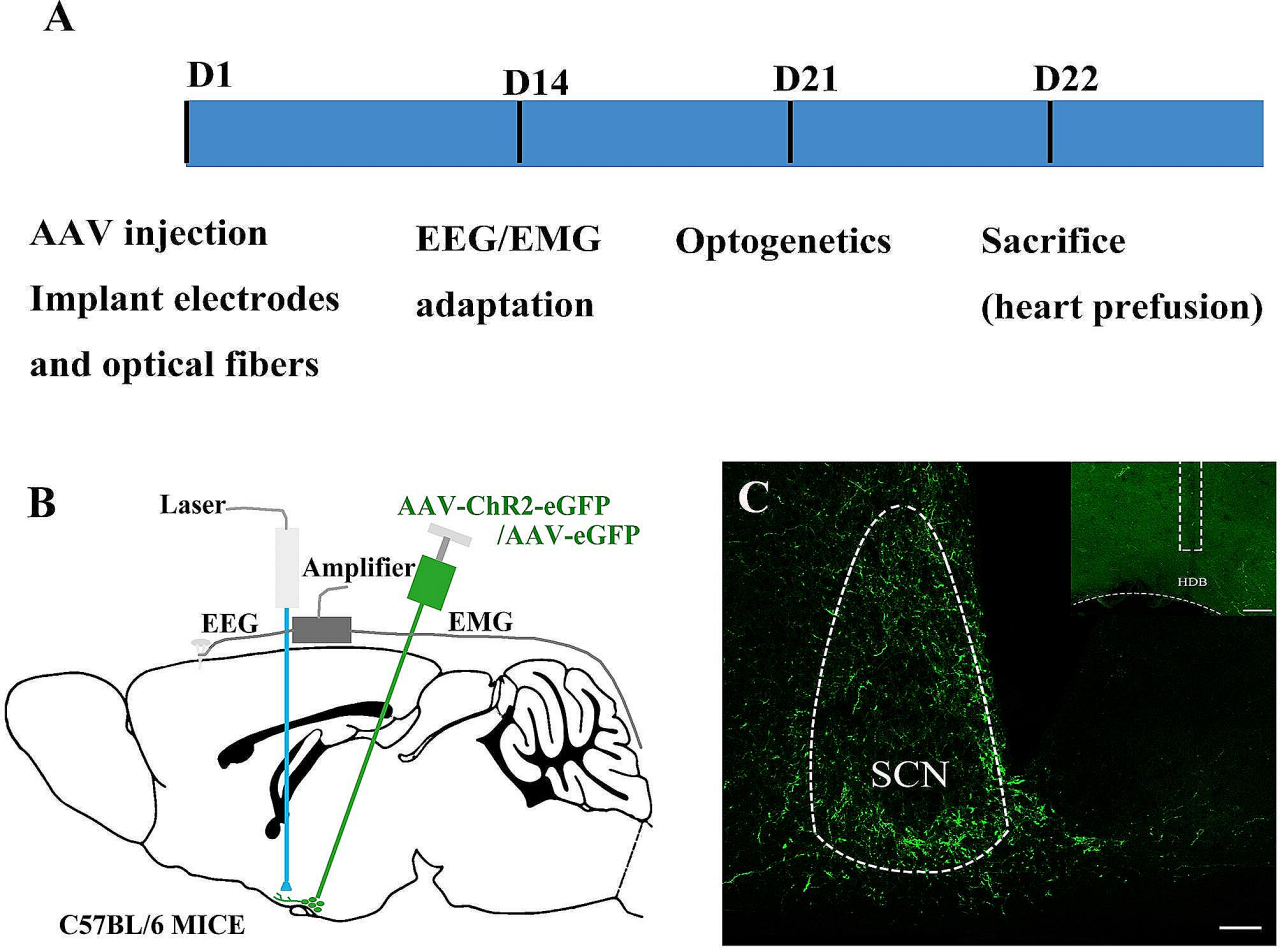
Optogenetic experiments of activating the SCN–HDB pathway. **A** A simple diagram of the experimental procedure. B Schematic diagram of virus injection, optical fiber implantation and EEG/EMG recording electrodes in C57BL/6J mice. **C** SCN and BF were injected with AAV-chR2-eGFP or the control virus AAV-GFP and implanted with optical fibers. The scale bar represents 150 μm

**Fig. 3:**
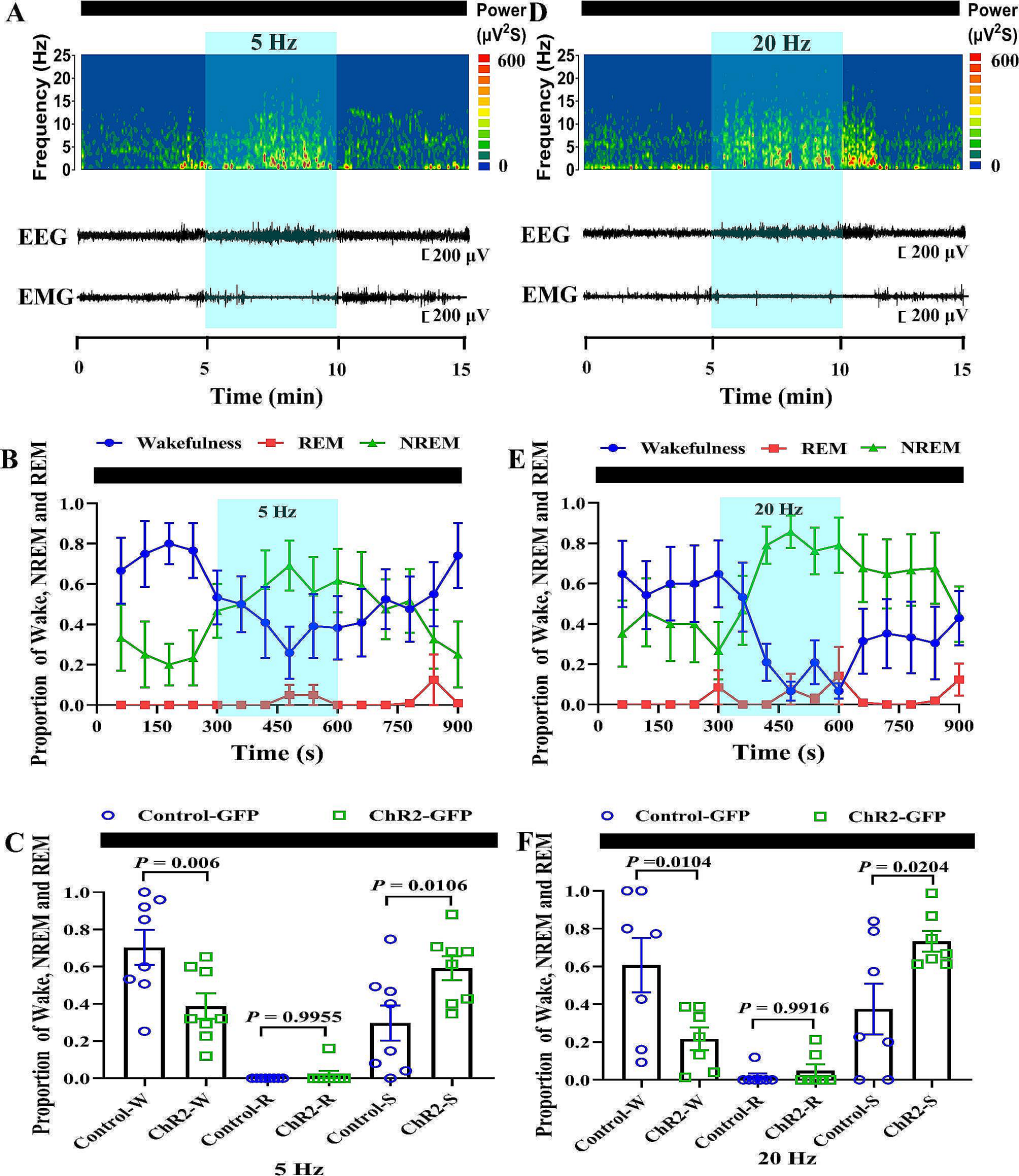
Optogenetic activation of the SCN-HDB pathway promotes NREM sleep during the dark phase. **A and D** An example trial of one of the optogenetic experiments. Shown are the EEG power spectrum, EEG traces and EMG trace of 900 s. The black bar represents the dark phase. The blue bar represents the period of laser stimulation (Left:10 ms per pulses, 5 Hz, 300 s; Right:10 ms per pulses, 20 Hz, 300 s). **B and E** Propotion of wake, NREM sleep, and REM sleep states before, during, and after 5–20 Hz laser stimulation during the dark phases. Blue shading indicates pulse laser stimulation of 300 s. **C and F** 5–20 Hz laser-induced change in the proportion of each states in AAV-ChR2-treated and AAV-eGFP-treated mice (*N* = 8 mice for 5 Hz, *N* = 7 mice for 20 Hz, One way ANOVA. Error bars represent ± s.e.m.) W means Wake; S means NREM sleep; R means REM sleep. The data in here came from 8 mice or 7 mice injected with control virus or ChR2 virus, and each data indicated the proportion of Wake, NREM and REM during the 300s period given 5–20 Hz blue light pulse stimulation

**Fig. 4:**
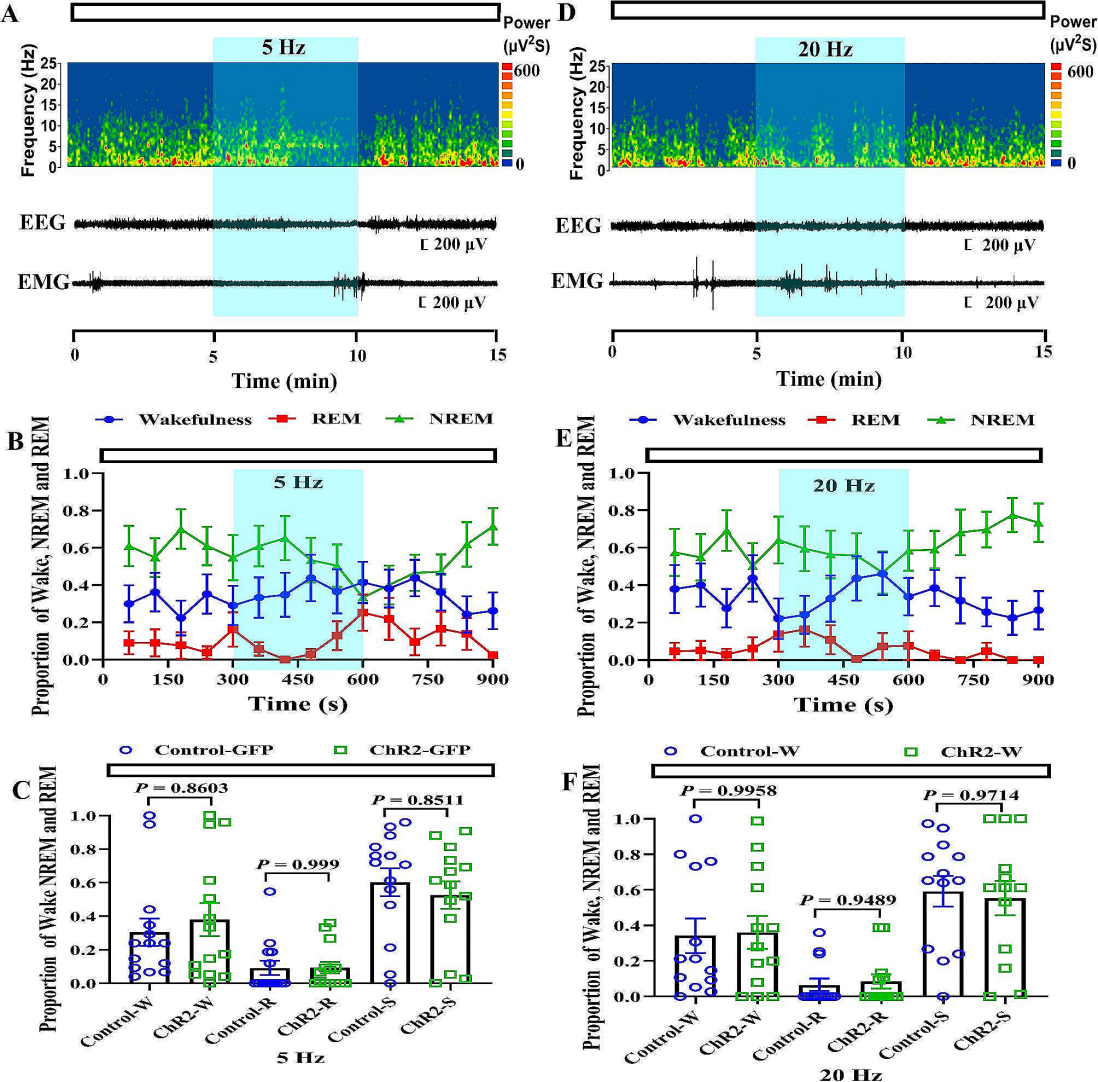
Optogenetic activation of the SCN-HDB pathway is not working during the light phase. **A and D** An example trial of one of the optogenetic experiments. Shown are the EEG power spectrum, EEG traces and EMG trace of 900 s. The white bar represents the light phase. The blue bar represents the period of laser stimulation (Left:10 ms per pulses, 5 Hz, 300 s; Right:10 ms per pulses, 20 Hz, 300 s). **B and E** Propotion of wake, NREM sleep, and REM sleep states before, during, and after 5–20 Hz laser stimulation during the light phases. Blue shading indicates laser pulse stimulation of 300 s. **C and F** 5–20 Hz laser-induced change in the proportion of each states in AAV-ChR2-treated and AAV-eGFP-treated mice (*N* = 14 mice for 5 Hz, *N* = 13 mice for 20 Hz, One way ANOVA). Error bars represent ± s.e.m. W means Wake; S means NREM sleep; R means REM sleep. The data in here came from 14 mice or 13 mice injected with control virus or ChR2 virus, and each data indicated the proportion of Wake, NREM and REM during the 300s period given 5–20 Hz blue light pulse stimulation

Although anatomical experiments confirmed the existence of the pathway, the function of this pathway in sleep-wake regulating remained unknown. Here, we activated the SCN**–**HDB pathway to clearly defined its function by using optogenetic manipulation. AAV2/9-hChR2(H134R)-EGFP was injected into the SCN. An optical fiber was implanted above the HDB. EEG/EMG electrodes were implanted in the skull at the same time (Fig. 2A-C). After 3 w, the pathway was activated by using blue light pulse stimulation of 1 Hz, 5 Hz (Figs. 3A-B and 4A-B),10 Hz and 20 Hz (Figs. 3D-E and 4D-E). The pulse stimulation was delivered in sessions of 300 s each (10 ms per pulse), and the stimulus intensity was 6–8 mW. The proportion of NREM sleep was significantly increased under only 5 Hz and 20 Hz but not 1 Hz and 10 Hz blue light pulse stimulation during the dark phase (Fig. 4C and F, Fig. S2A-B and Fig.S3C-D). During the light phase, there were no significant differences in NREM sleep and wakefulness times (Fig. 3C and F and Fig.S3A-B).

### Chemogenetic activation of the SCN–HDB pathway promotes NREM sleep during the dark phase

**Fig. 5:**
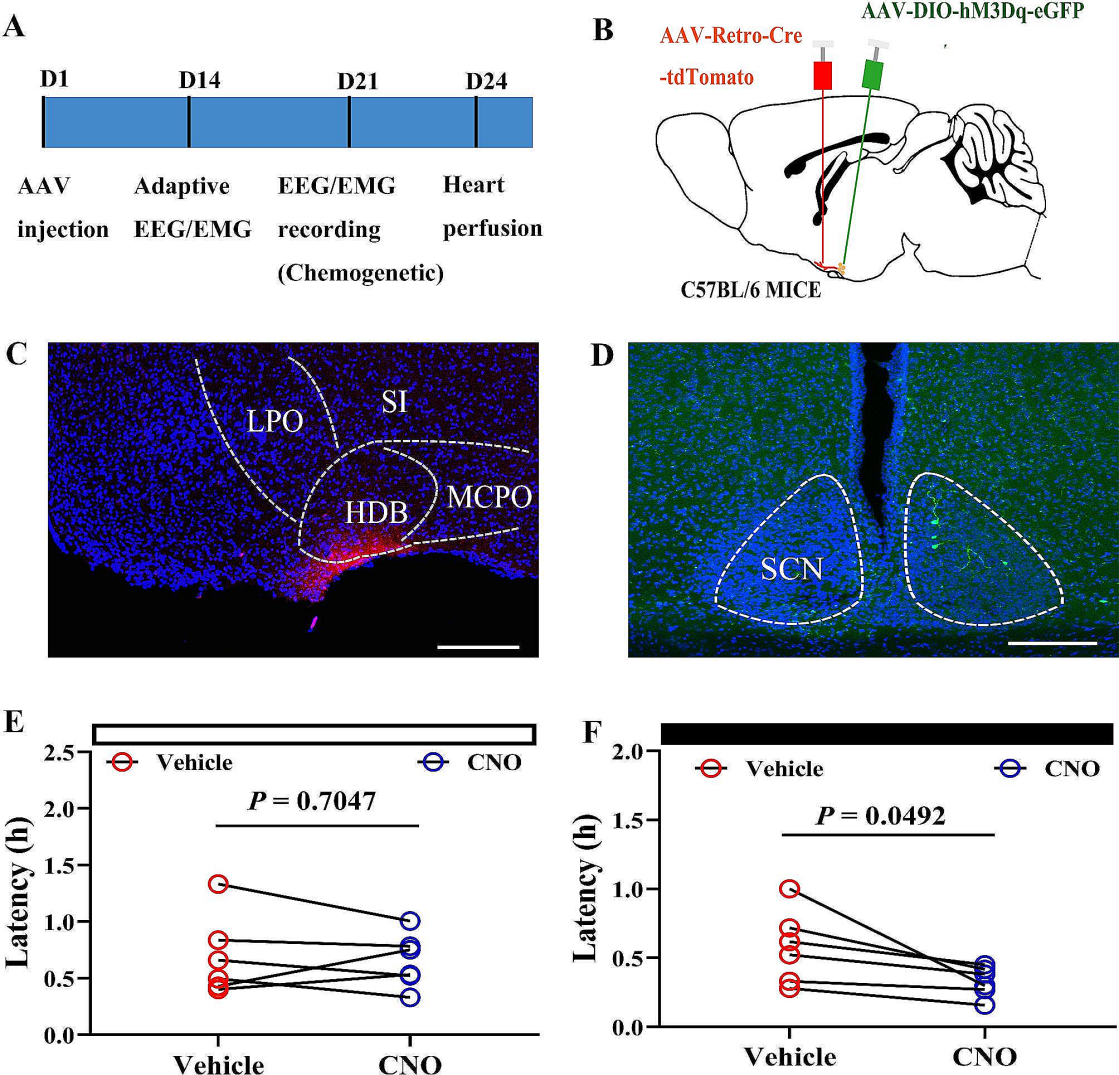
Chemogenetic activation of the SCN–HDB pathway shorten the lantency during the dark phase. **A** A simple diagram of the chemogenetic experimental procedure. **B** Schematic diagram of virus injection in C57BL/6 mice. **C** The HDB was injected with AAV2/2-Retro-tdTomato-iCre. The scale bar represents 100 μm. **D** The SCN was injected with AAV-DIO-hM3Dq-eGFP or AAV-DIO-eGFP. SCN neurons projecting to the HDB express eGFP. The scale bar represents 100 μm. **E** The lantency of the NREM sleep (time form light onset until first NREM sleep) during the light phase (*N* = 6 mice, *P < 0.05, vehicle-hM3Dq vs. CNO-hM3Dq, paired T test). **F** The lantency to NREM sleep (time from light off until first NREM sleep) during the dark phase (*N* = 6 mice, *P < 0.05, vehicle-hM3Dq vs. CNO-hM3Dq, paired T test). Vehicle-hM3Dq: SCN was injected to express eGFP instead of hM3Dq (i.p. NACL); CNO-hM3Dq: SCN was injected to express hM3Dq (i.p. CNO); CNO-GFP: SCN was injected to express eGFP instead of hM3Dq (i.p. CNO)

The optogenetic results showed that activation of the SCN**–**HDB pathway promotes NREM sleep during the dark phase. To further verify the results, chemogenetic methods were used. AAV2/2-Retro-tdTomato-iCre into the HDB and injected AAV2/9-DIO-hM3D(Gq)-eGFP were injected into the SCN (Fig. 5A-D). By analyzing the lantency of NREM sleep, we found that the administration of CNO significantly shorten sleep lantency compared to the control group during the dark phase (Fig. 5E-F).

**Fig. 6:**
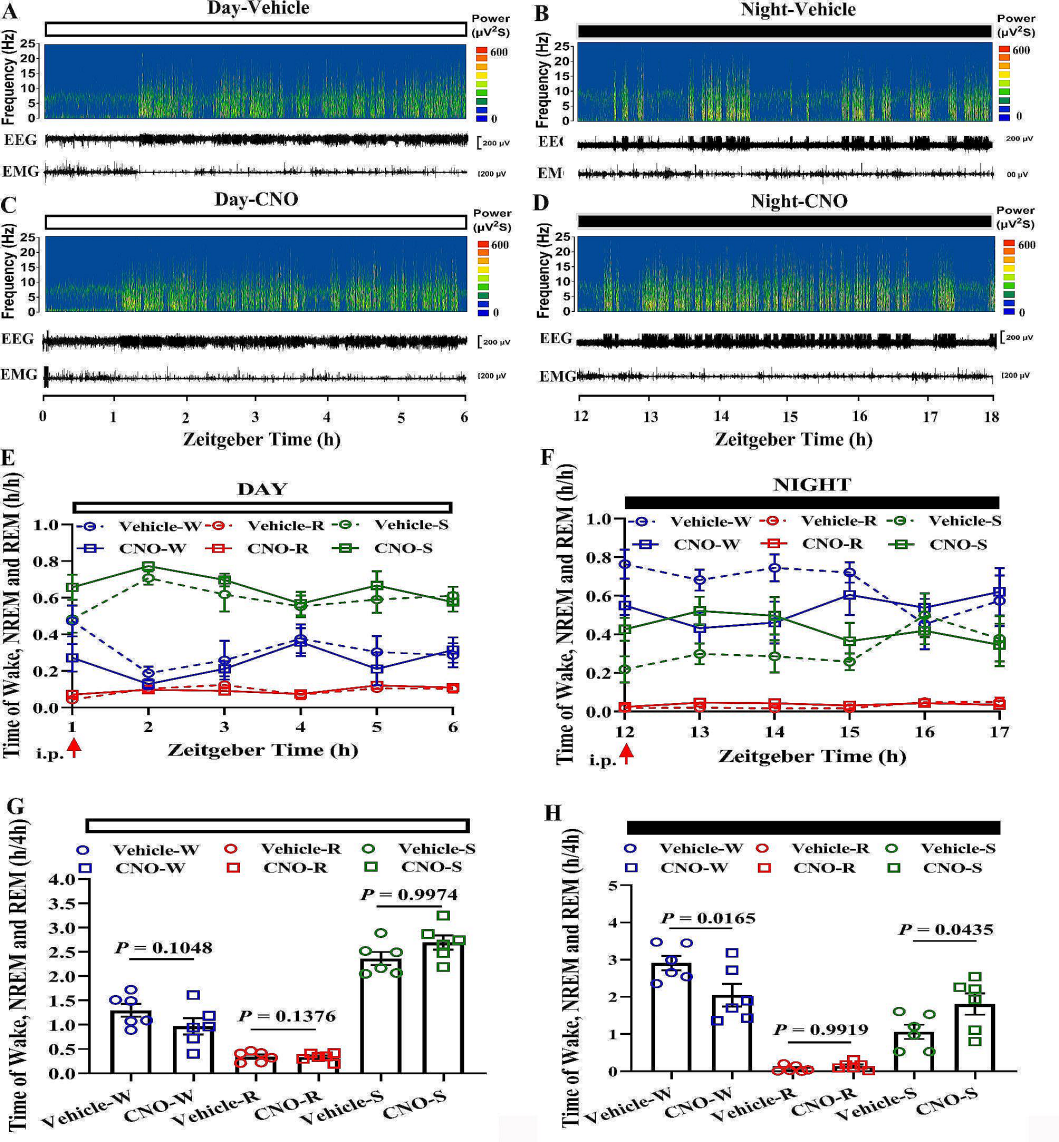
Chemogenetic activation of the SCN–HDB pathway promotes NREM sleep during the dark phase. **A-D** The example trial of the chemogenetic experiments. The EEG power spectrum, EEG traces and EMG traces after CNO injection are shown. The white bar represents the vehicle and CNO group in the light phase, and the black bar represents the vehicle and CNO group in the dark phase. **E and F** After CNO injection, EEG and EMG were recorded continuously during the light and dark phase. Time spent in wakefulness, NREN sleep and REM sleep was counted. **G** and **H** The total durations of wakefulness, NREM sleep, and REM sleep in a 4 h period in the vehicle-hM3Dq group and CNO-hM3Dq group during the light and dark phase (*N* = 6 mice, vehicle-hM3Dq vs. CNO-hM3Dq, One way ANOVA). W means Wake; S means NREM sleep; R means REM sleep

**Fig. 7:**
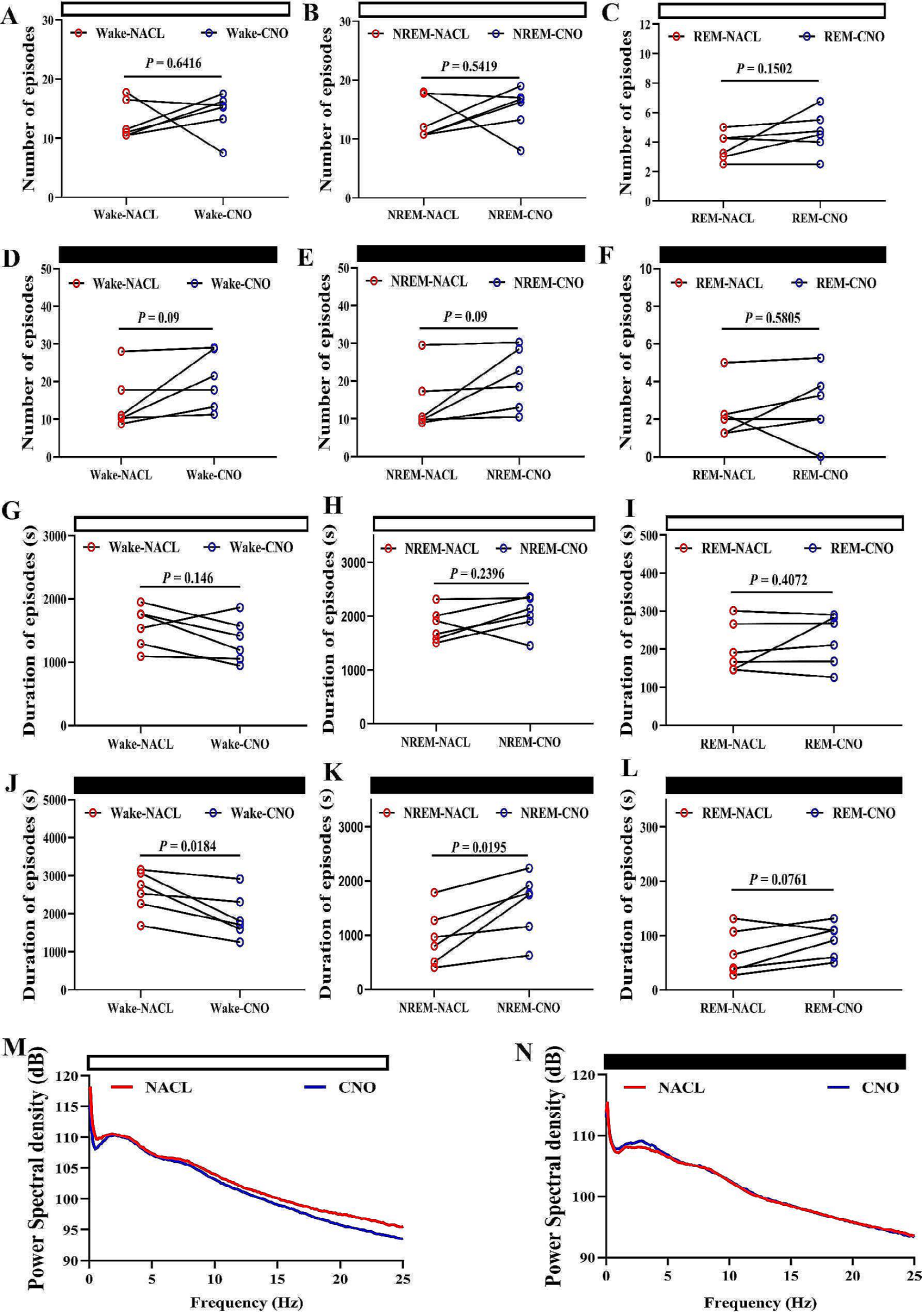
Chemogenetic activation of the SCN–HDB pathway increases duration of episodes of NREM sleep during dark phase. **A-C** The statistical graph of number of episodes of wakefulness, NREM sleep, REM sleep during light phase (*N* = 6 mice, NACL-hM3Dq vs. CNO-hM3Dq, Paired T test). **D-F** The statistical graph of number of episodes of wakefulness, NREM sleep, REM sleep during dark phase (*N* = 6 mice, NACL-hM3Dq vs. CNO-hM3Dq, Paired T test). **G-I** The statistical graph of duration of episodes of wakefulness, NREM sleep, REM sleep during light phase (*N* = 6 mice, NACL-hM3Dq vs. CNO-hM3Dq, Paired T test). **J-L** The statistical graph of duration of episodes of wakefulness, NREM sleep, REM sleep during dark phase (*N* = 6 mice, NACL-hM3Dq vs. CNO-hM3Dq, Paired T test). **M** The statistical graph of PSD during light phase. **N** The statistical graph of PSD during dark phase

Furthermore, the time of NREM sleep-promoting effect lasted for 4 h (Fig. 6A-D). The time of NREM sleep significantly increased in the 4 h after CNO administration during the dark phase (Fig. 6E-H). There was no significant effect on the sleep**–**wake cycles during the light phase (Fig. 6C). Meanwhile, the numbers of episode, duration of episodes and PSD were also analyzed (Fig. 7). No changs in the number of episodes of wakefulness, NREM sleep, REM sleep during light or dark phase (Fig. 7.A-F). Only the duration of episodes of wakefulness, NREM sleep, REM sleep has changed during the dark phase (Fig. 7.G-L). In addition, the overall PSD was been a slight change at 0.5–4.5 Hz during night phase, which is characteristic of NREM sleep. From these results, we can see that activating this pathway can increase the effect of sleep on the structure of sleep wake. It was not due to increasing the number of episodes of NREM sleep, but to lengthening the duration of episodes each NREM sleep.

## Discussion

In recent years, the circuitry and function of brain regions involved in the sleep–wake regulation have been characterized by different viral tools [20, 21]. In this study, we verified the direct innervation of the BF and the SCN. First, we confirmed that the HDB subregion is the downstream target nucleus of the SCN by retrograde tracing. Furthermore, both optogenetic and chemogenetic activation of the SCN**–**HDB pathway promotes NREM sleep during the dark phase. These findings elucidate the role of the SCN**–**HDB pathway in sleep**–**wake regulation.

### The HDB is downstream of the SCN

Previous studies have shown that the BF is the primary projection targets of the SCN [15, 16]. However, the function of the SCN**–**BF pathway has not been reported. Our experiment used the anterograde AAV and retrograde tracer to confirm that the SCN projects to the HDB (Fig. 1 and Fig.S1). The HDB contains cholinergic and GABAergic neurons which located adjacent to the VLPO, a sleep-promoting center; these two regions may be functionally related [22, 23]. In our previous studies, we found that anesthetics DEX can affect neuronal activity in HDB [24]. These findings suggest that the HDB nucleus may play a role in sleep–wake regulating. However, detailed studies on the involvement of HDB in the sleep**–**wake regulation is still insufficient. Our study may also suggest a possible link between this role.

### Optogenetic activation of the SCN–HDB pathway promotes NREM sleep during the dark phase

In this study, optogenetic activation of the SCN**–**HDB pathway could promote NREM sleep (Figs. 3 and 4). Light pulses stimulation at a frequency of 5–20 Hz could promote NREM sleep during the dark phase (Fig. 4, Fig.S2). A possible explanation is that the mice are nocturnal and therefore mainly active during the dark phase. Sleep pressure accumulates during wakefulness and dissipates during sleep [25]. Since the mice mostly awake during the dark phase, it may have accumulated more sleep pressure, making it easier to fall asleep. However, it spend most of their time asleep during the light phase, accumulating less sleep pressure and making it difficult to further increase their NREM sleep. According to the statistical results, 20 Hz pulse stimulation can cause a change of 40%, but 5 Hz frequency can only cause a change of nearly 30%. The high frequency pulse of 20 Hz can play this role more obviously. This may be caused by the fact that this high frequency pulse is closer to the normal physiological frequency. Meanwhile, our results showed that 1 Hz and 10 Hz light stimulation had no effect on NREM sleep. Since we ignored the sleep state of the mice at the beginning of the optogenetic experiment, this may affect the results of the experiments. Another possible reason is that the experimental sample size is not enough. Furthermore, we found that within that 300 s showed a significant increase in NREM sleep (Fig. 4). Previous research mostly used 120 s periods of light pulse stimulation [26, 27]. However, there was no significant difference between the first 120s and the remaining 300 s. The possible reason is that the experimental sample size is not enough.

Several neurotransmitters are expressed in the SCN, almost all SCN neurons are GABAergic [28]. Moreover, GABA neurons in the SCN nucleus are co-located with a variety of neuropeptides, and the overall neuronal inhibition cannot explain the problem. In addition, there was a lack of inhibition experiments in our experiment. Since virus injection has the limitation of injection site, and can only inhibit neurons at the injection site, but not all neurons in the SCN nucleus. Therefore, in the next step of our experiment, we will further focus on specific neuropeptide neurons to detect their specific functions.

### Chemogenetic activation of the SCN–HDB pathway promotes NREM sleep during the dark Phase

In this study, activation of the SCN**–**HDB pathway by chemogenetic experiments produced significant changes in NREM sleep but not REM sleep (Figs. 5 and 6). From the chemogenetic experimental results, it can be seen that this sleep-promoting effect occurs within 4 h. We also compared the effective time that other researchers used chemogenetic experiments. Most were found to be concentrated in 2–6 h, but there were also those that lasted 12 h or more [29, 30]. The reason of lasting 4 h in our experiment may be its own homeostasis regulation of the sleep**–**wake states. Therefore it is impossible to stay in a certain state for a particularly long time. After a certain period of time, it may return to its original state due to other compensatory effects.

Previous studies have shown that the BF is a key nucleus that regulates NREM sleep [31]. Cholinergic, glutamatergic and parvalbumin-positive GABAergic (GABA^PV+^) neurons in the BF can reduce NREM sleep, while GABA^SOM+^ neurons can promote NREM sleep [32]. Neither has any effect on REM sleep, which is consistent with our experimental results [19]. Studies of REM sleep mostly focus on neural circuits of the pons, and more generally, the brainstem, which are required for REM sleep [33–35]. Moreover, the hypothalamus also plays a role in REM sleep, as reported in recent studies [36, 37]. It can be seen that SCN dominates the downstream nucleus of BF, and thus participates in the regulation of sleep wake function.

## Conclusion

Our findings reveal that the SCN–HDB pathway participates in NREM sleep regulation and provides direct evidence of a novel SCN-related pathway involved in sleep–wake states regulation.

### Electronic supplementary material

Below is the link to the electronic supplementary material.

Supplementary Material 1

Supplementary Material 2

## Data Availability

The data are available from the corresponding author upon reasonable request.

## Declarations

### Generative AI in scientific writing

The authors do not use generative artificial intelligence (AI) and AI-assisted technologies in the writing process.
