# Supplementary material for: Efferent pathways from the suprachiasmatic nucleus to the horizontal limbs of diagonal band promote NREM sleep during the dark phase in mice
Source: BMC Neurosci. 2024 Jul 22;25:34. doi: 10.1186/s12868-024-00881-0 (PMC11265431; doi:10.1186/s12868-024-00881-0)
Supplement: Supplementary file 1 — Supplementary Material 1 [file 12868_2024_881_MOESM1_ESM.docx]

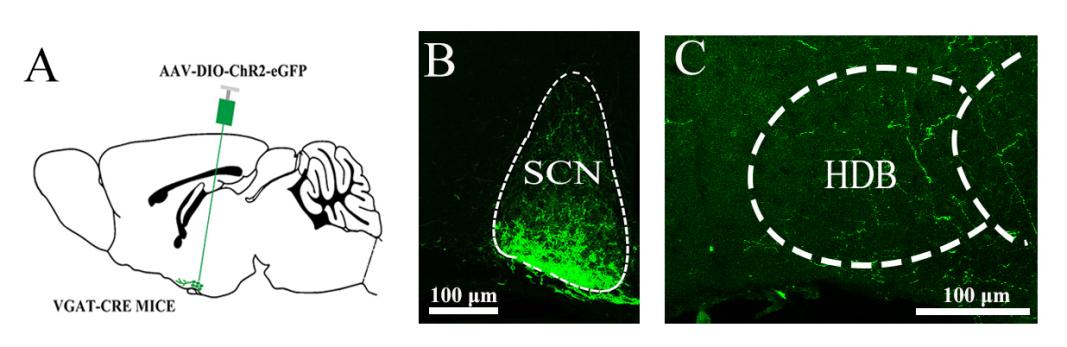


**Fig. S1** **The SCN projects to the HDB.** **A** Schematic of the injection of the AAV-DIO-chR2-eGFP into the SCN of Vgat-Cre mice. **B** Localization of AAV-DIO-chR2-eGFP in the SCN. The scale bar represents 100 μm. **C** The projection of nerve fiber terminals in the HDB subregion of BF. The scale bar represents 100 μm.


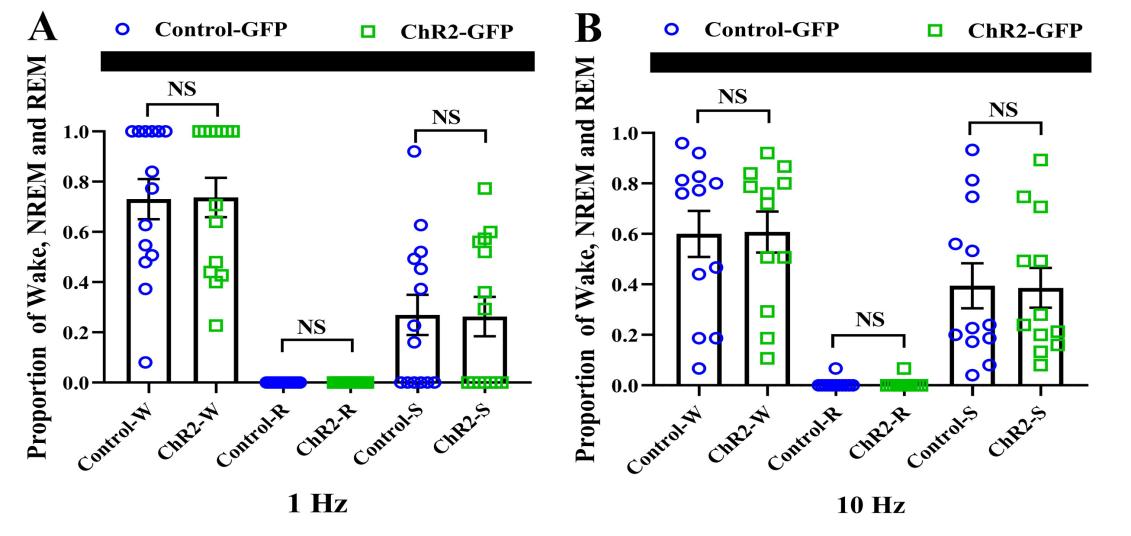


**Fig. S2** **1 Hz and 10 Hz** **pulse stimulation did not affect sleep-wake cycles during the night phase.** **A and B** 1 Hz or 10 Hz blue pulse stimulation laser-induced change in the proportion of each state in AAV-ChR2-treated and AAV-eGFP-treated mice (N = 8 mice, NS means no significant differences). Error bars represent ± s.e.m. W means Wake; S means NREM sleep; R means REM sleep.


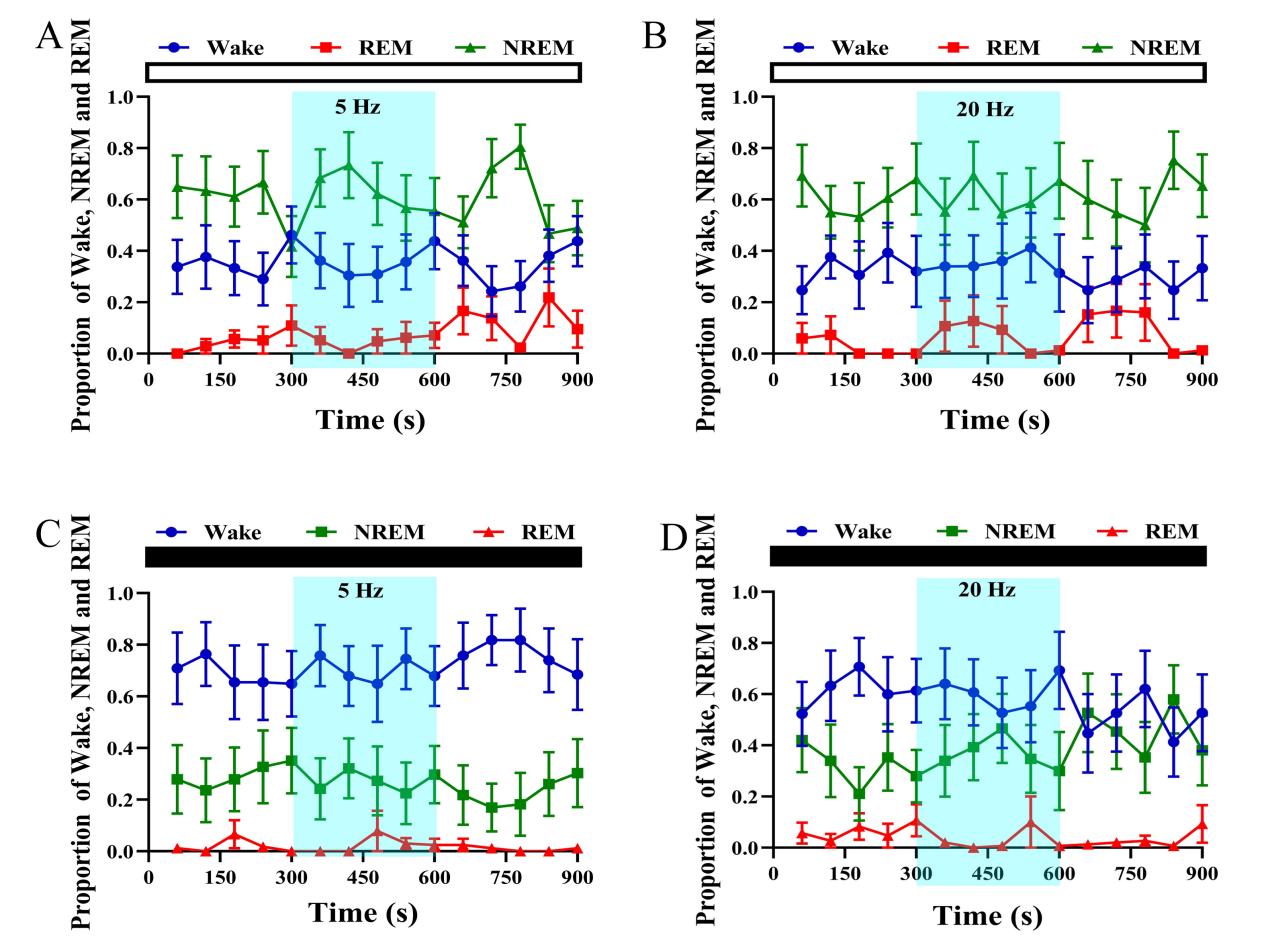


**Fig. S3** **5 Hz and 20 Hz pulse stimulation in GFP group did not affect sleep-wake cycles during the day/night phase**

**A and B** Propotion of wake, NREM sleep, and REM sleep states before, during, and after 5 Hz or 20 Hz laser stimulation during the day phases of GFP group. Blue shading indicates pulse laser stimulation of 300 s. **C and D** Propotion of wake, NREM sleep, and REM sleep states before, during, and after 5 Hz or 20 Hz laser stimulation during the dark phases of GFP group. Blue shading indicates pulse laser stimulation of 300 s.
